# The Impact of Juvenile Coxsackievirus Infection on Cardiac Progenitor Cells and Postnatal Heart Development

**DOI:** 10.1371/journal.ppat.1004249

**Published:** 2014-07-31

**Authors:** Jon Sin, Jenna M. Puccini, Chengqun Huang, Mathias H. Konstandin, Paul E. Gilbert, Mark A. Sussman, Roberta A. Gottlieb, Ralph Feuer

**Affiliations:** 1 Donald P. Shiley BioScience Center, San Diego State University, San Diego, California, United States of America; 2 The Integrated Regenerative Research Institute (IRRI) at San Diego State University, Cell & Molecular Biology Joint Doctoral Program, Department of Biology, San Diego State University, San Diego, California, United States of America; 3 San Diego State Heart Institute, San Diego State University, San Diego, California, United States of America; 4 Department of Psychology, San Diego State University, San Diego, California, United States of America; University of Utah, United States of America

## Abstract

Coxsackievirus B (CVB) is an enterovirus that most commonly causes a self-limited febrile illness in infants, but cases of severe infection can manifest in acute myocarditis. Chronic consequences of mild CVB infection are unknown, though there is an epidemiologic association between early subclinical infections and late heart failure, raising the possibility of subtle damage leading to late-onset dysfunction, or chronic ongoing injury due to inflammatory reactions during latent infection. Here we describe a mouse model of juvenile infection with a subclinical dose of coxsackievirus B3 (CVB3) which showed no evident symptoms, either immediately following infection or in adult mice. However following physiological or pharmacologically-induced cardiac stress, juvenile-infected adult mice underwent cardiac hypertrophy and dilation indicative of progression to heart failure. Evaluation of the vasculature in the hearts of adult mice subjected to cardiac stress showed a compensatory increase in CD31^+^ blood vessel formation, although this effect was suppressed in juvenile-infected mice. Moreover, CVB3 efficiently infected juvenile c-kit^+^ cells, and cardiac progenitor cell numbers were reduced in the hearts of juvenile-infected adult mice. These results suggest that the exhausted cardiac progenitor cell pool following juvenile CVB3 infection may impair the heart's ability to increase capillary density to adapt to increased load.

## Author Summary

Coxsackievirus B (CVB) is a significant human pathogen, causing myocarditis, aseptic meningitis and encephalitis. The lasting consequences of juvenile CVB infection on the developing host have yet to be adequately inspected. Here, we show that CVB efficiently infected juvenile cardiac progenitor cells both in culture and the young heart. Furthermore, we describe a mouse model of juvenile infection with a subclinical dose of CVB which showed no symptoms of disease into adulthood. However following physiological or pharmacologically-induced cardiac stress, juvenile-infected mice underwent cardiac hypertrophy and dilation indicative of progression to heart failure. These results suggest that mild CVB infection in the young host may impair the ability of the heart to adapt to increased load leading to pathological remodeling later in adult life.

## Introduction

Coxsackieviruses (CV) are common human pathogens that typically cause a self-limited infection and mild symptoms such as fever, rash, and upper-respiratory complications. Though CV can also cause severe inflammatory diseases including myocarditis, a disease that can lead to dilated cardiomyopathy [1], [2], [3], the manifestation of a cardiac disease phenotype has been documented to be extremely rare (∼5% of infected patients) [4]. Collapse and death of young individuals during exertion can result from catastrophic dysfunction of the electrical pathways in the heart associated with unrevealed CV infection [5], [6]. Additionally, 70–80% of individuals with end-stage idiopathic dilated cardiomyopathy have detectable levels of CV RNA in the myocardium without any history of antecedent viral myocarditis [7], [8], [9]. These findings raise the possibility that mild infection with CV can cause subtle but lasting injury, although it is unclear whether such enduring damage is immune-mediated or due to virus-mediated cytopathic effects. Previous studies suggest that coxsackievirus B3 (CVB3) may exhibit unique tropism for undifferentiated cells such as neural and hematopoietic progenitor cells thereby altering cell lineage commitment or diminishing their restorative capacity [10], [11], [12], [13], [14], [15], [16], [17]. Infection of progenitor cells may also enhance virus dissemination in a process referred to autophagosome-mediated exit without lysis (AWOL) [18], [19]. Based on these observations, we hypothesized that CVB3 also infects cardiac progenitor cells which might lead to long-term consequences for heart development and function. Of note, the mechanistic basis and causal link between juvenile CVB3 infection and adult-onset dilated cardiomyopathy has not been previously inspected.

The heart was previously viewed as a terminally differentiated organ predominantly comprised of a fixed number of non-renewable cardiomyocytes. Recently, a distinct population of resident cardiac progenitor cells (CPCs) was identified that challenged the notion of a heart without regenerative capacity. These CPCs were described as exhibiting large nuclei, scant cytoplasm, and hematopoietic markers such as CD117 (c-Kit) and Sca-1 [20], [21], [22], [23]. c-kit^+^ cells isolated from the heart and grown in culture are capable of differentiating into all four cell types of the cardiac lineage which include cardiomyocytes, smooth muscle cells, endothelial cells, and fibroblasts. CPCs also play a beneficial role in adult cardiac repair, and the local injection of isolated CPCs have been shown to preserve myocardial muscle mass and reduce scar formation after experimental myocardial infarction in mice [24].

Due to their role in cardiac development as well as in cardiac maintenance, a perturbation of the CPC pool due to infection or other environmental factors could conceivably have deleterious effects on the developing heart. Previously, Huang et al demonstrated that in mouse pups, exposure to the chemotherapeutic drug doxorubicin before postnatal day 21 resulted in a decreased number of CPCs due to early senescence. Although doxorubicin-treated animals exhibited normal cardiac function at 8 weeks of age, endurance exercise led to the development of pathological hypertrophy and fibrosis [25]. This model seems to mirror the late consequences of childhood doxorubicin-induced cardiotoxicity observed in humans which manifests in weakening of the myocardium, an attenuation of cardiac pump performance, and progression towards congestive heart failure. Since previous studies described preferential CVB3 infection of progenitor cells in the CNS [10], [11], [15], [16], [26] and bone marrow [17], we investigated the effect of CVB3 on CPCs in the developing heart. We established a mouse model of juvenile CVB3 infection and observed that not only did CVB3 productively infect CPCs, but infection caused a sustained reduction in the CPC pool into adulthood. We suggest that the exhaustion of CPCs within the heart predisposed the adult animal to stress-induced cardiac hypertrophy, left ventricular dilation, and the accumulation of fibrotic tissue – features considered to be the early stages of dilated cardiomyopathy. These results provide evidence that a mild CVB3 infection may predispose the heart to pathologic remodeling later in adult life.

## Results

### CVB3 infected cardiac-derived c-kit^+^ cells grown in culture

**Figure 1:**
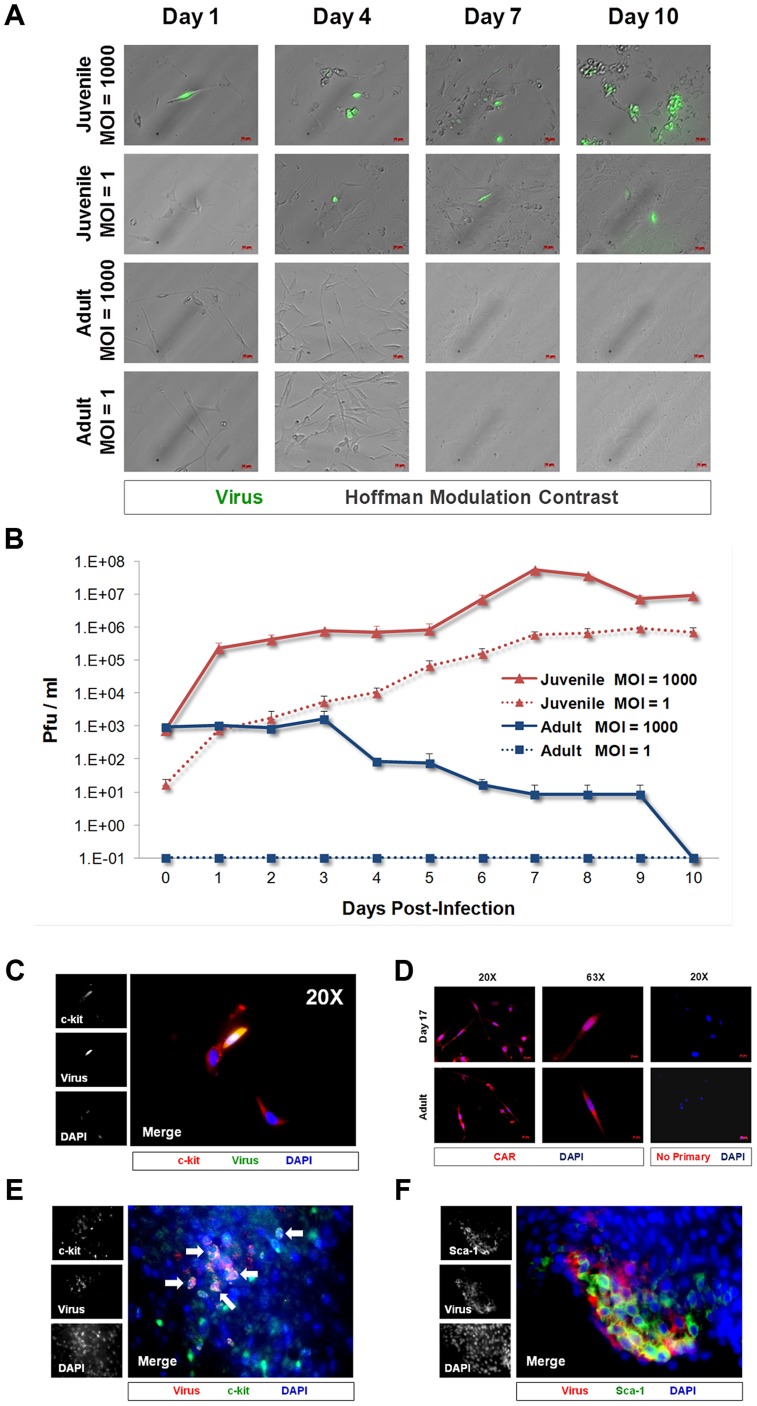
CVB3 productively infected juvenile cardiac-derived c-kit+ cells grown in culture and in the juvenile heart. c-kit^+^ cells were isolated from the heart of juvenile or adult mice and expanded in culture. *(A)* Cell cultures were infected with eGFP-CVB3 (moi = 1 or 1000), and infected cultures were followed by fluorescence microscopy for 10 days. *(B)* Viral titers were determined from supernatants of infected cell cultures over 10 days PI. *(C)* Juvenile c-kit^+^ cells infected with eGFP-CVB3 (green) were fixed in 2% paraformaldehyde and stained by immunocytochemistry using an antibody against c-kit (red). *(D)* Juvenile and adult c-kit^+^ cells were stained by immunocytochemistry using an antibody against coxsackie and adenovirus receptor (CAR, red). *(E)* Three day-old mice were infected with eGFP-CVB3 (10^5^ pfu IP) or mock-infected, and hearts were isolated at 2 days PI. Paraffin-embedded sections of heart tissue were deparaffinized and stained using an antibody against c-kit (red) and GFP (virus, green). Many virus-infected c-kit^+^ cells were observed in heart sections (white arrows). *(F)* Also, Sca-1^+^ cells (green) in heart tissue were shown to be infected with eGFP-CVB3 (red). Representative images of three infected mice are shown.

We previously determined that proliferating neural stem and progenitor cells grown in the subventricular zone of the neonatal brain or grown in culture were preferentially targeted by CVB3 [10], [11], [16], [14], [26]. Furthermore, CVB3 reduced the levels of neurogenesis and altered brain development in a neonatal model of viral infection [15]. To investigate if CVB3 infected cardiac progenitor cells (CPCs) in a similar fashion, c-kit^+^ cells were isolated from juvenile (Day 17 post-birth) or adult mice and cultured in chamber slides with complete CPC growth medium. Cultured cardiac derived c-kit^+^ cells were infected with eGFP-CVB3 at a multiplicity of infection (MOI) of 1 or 1000. The production of infectious virus was determined by plaque assay or viral protein expression (eGFP) utilizing fluorescence microscopy up to 10 days post-infection (PI) (***Figure 1***). Our results showed that juvenile c-kit^+^ cells expressed high levels of viral protein (eGFP) at low and high MOI (***Figure 1A***). Also, juvenile c-kit^+^ cells produced high amounts of infectious virus (***Figure 1B***). In contrast, adult c-kit^+^ cells failed to express detectable levels of viral protein or significant amounts of infectious virus. These differences are consistent with clinical studies suggesting that infants are much more susceptible to CVB3 infections than adults [27], [28], [29]. As the course of infection progressed, a majority of juvenile c-kit^+^ cells exhibited cytopathic effects, detached from the surface of the culture dish and fragmented into apoptotic bodies. Nevertheless, some juvenile c-kit^+^ cells survived initial infection and continued to proliferate in culture.

Colocalization of c-kit and virus protein expression in cultured CPCs infected with eGFP-CVB3 was identified by immunofluorescence microscopy (***Figure 1C***). Additionally, we observed a population of low eGFP-expressing juvenile c-kit^+^ cells which remained morphologically normal. At later time points, eGFP was not detected, and infected cultures developed a flattened morphology consistent with differentiation (data not shown). In contrast, adult c-kit^+^ cells produce little to no infectious virus and appeared resistant to infection. Both juvenile and adult c-kit^+^ cell populations expressed high levels of coxsackie and adenovirus receptor (CAR) (***Figure 1D***), suggesting that virus receptor levels were not responsible for the differential susceptibility to infection. These results parallel our findings regarding infection in the neonatal CNS whereby CAR expression is widespread, yet the tropism of the virus is specific for proliferating NPSCs early after infection [10], [11]. The presence of CAR on adult c-kit^+^ cells suggests that factors other than CAR expression may ultimately be responsible for increased susceptibility of juvenile CPCs to CVB3 infection.

Next, to determine if CVB3 infected CPCs *in vivo*, immunostaining was performed on the hearts of mouse pups infected with eGFP-CVB3 (10^7^ pfu IP). Hearts were harvested 2 days PI and paraffin-embedded for histological analysis. Viral protein expression (eGFP) colocalized with c-kit and Sca-1, indicating infection of CPCs in the juvenile heart (***Figure 1E*** and ***Figure 1F***, respectively). Interestingly, viral protein expression was not observed in the surrounding myocardium, suggesting that CPCs were preferentially targeted for infection in the juvenile heart.

### Establishing a mouse model of subclinical CVB3 infection to examine CPC alterations in the heart

**Figure 2:**
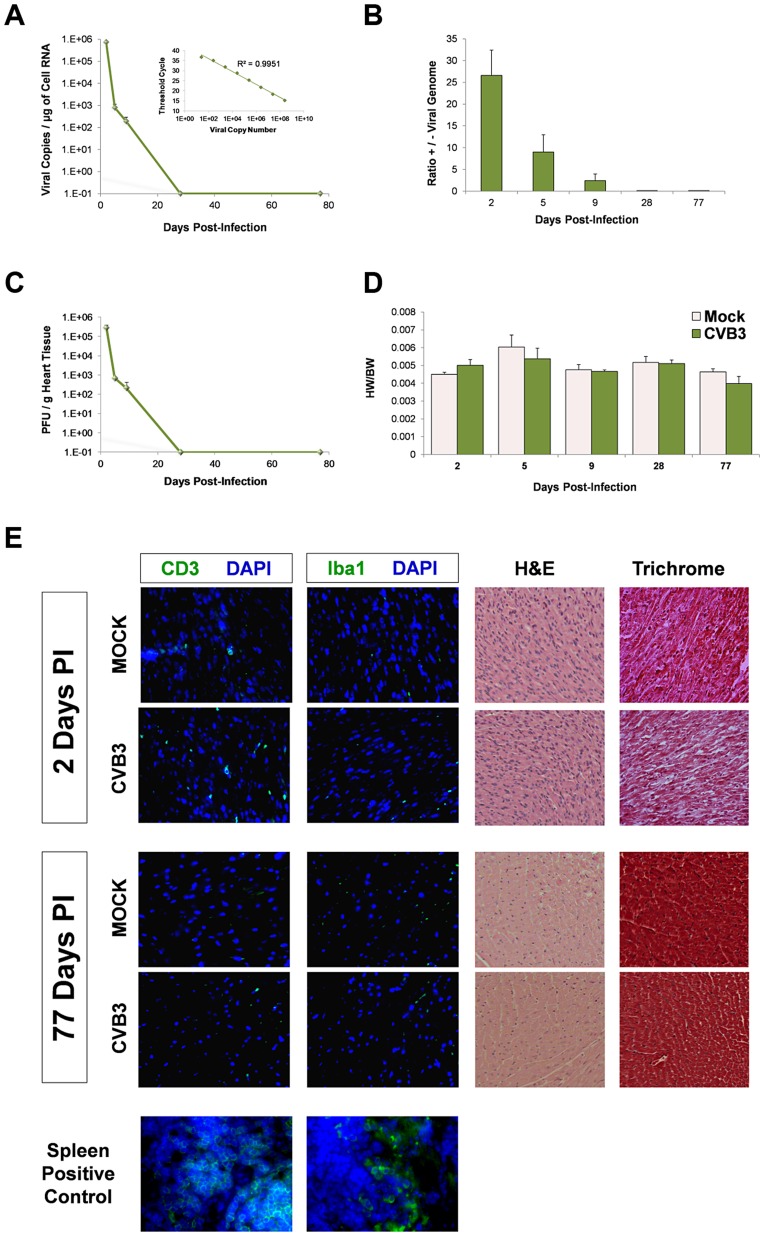
No discernible cardiac hypertrophy or immunopathology in the adult heart following juvenile CVB3 infection. Three day-old mice were infected with eGFP-CVB3 (10^5^ pfu IP) or mock-infected, and inspected for viral titers over time. *(A)* Viral copies in the heart were determined by quantitative real time RT-PCR. The R^2^ value (0.997) was determined by plotting C_T_ values versus viral copy number (upper right corner). An R^2^ value of 1.00 indicates that all data points lie perfectly on the graphed data set line. *(B)* The ratio of positive-sense to negative-sense viral genome was calculated over time in the heart. *(C)* Viral titers in the heart were determined using standard plaque assay on HeLa cells. *(D)* Using two-way ANOVA statistical analysis, no differences in heart weight to body weight ratios were observed between infected (day 2, n = 10; day 6, n = 8; day 9, n = 5; day 28, n = 12; day 77, n = 8) and mock-infected mice (day 2, n = 19; day 6, n = 8; day 9, n = 5; day 28, n = 6; day 77, n = 6) up to 77 days PI. *(E)* Heart sections from mice immunostained for T cells (CD3) and macrophages (Iba1) at 2 and 77 days PI showed the relatively low levels of inflammatory cells in the myocardium. Also, heart sections were inspected for histopathology by hematoxylin & eosin (H&E), or Masson's trichrome staining. H&E staining confirmed normal myofibrillar arrangements and Masson's trichrome staining showed an absence of fibrosis following infection. Representative images of three infected or mock-infected mice are shown. Also, spleen positive controls for both CD3 and Iba1 staining are shown.

To study sublethal juvenile infection, mice were infected with 10^7^ pfu GFP-CVB3 at postnatal day 3 (***Figure 2***). This inoculum of virus was well-tolerated, and infected mouse pups showed no overt health abnormalities. Suckling and weight gain in infected pups was similar to the mock-infected control mice. Despite the absence of overt illness, a large amount of infectious virus and viral genomic RNA was detectable in the heart up to 9 days PI, confirming cardiac tropism of the virus (***Figure 2A***) as shown previously in the adult mouse model by other groups [30], [31], [32]. Also, a stepwise reduction in the ratio of positive to negative sense strand viral genome was observed over time in the heart (***Figure 2B***), similar to previous studies describing CVB3 infection in the neonatal CNS [33]. Viral titers in the heart were determined over time by standard plaque assay on HeLa cells and closely paralleled results using quantitative real time RT-PCR for positive sense viral genome (***Figure 2C***). No significant difference in the heart weight to body weight ratio (HW/BW) was observed between CVB3 and mock-infected animals at any time point (***Figure 2D***; two-way ANOVA). Immunofluorescence for CD3 and Iba-1 (T-cells and macrophages, respectively) revealed no significant immune cell infiltration following CVB3 infection in the heart of juvenile mice given a sublethal dose of virus (***Figure 2E***). At 2 days PI, hematoxylin-eosin (H&E) staining showed that both infected and mock-infected mice showed normal arrangement of myofibrils. Also, Masson's trichrome staining revealed no myocardial scarring during the acute phase of infection.

### Increased Sca-1 expression in the infected juvenile myocardium

**Figure 3:**
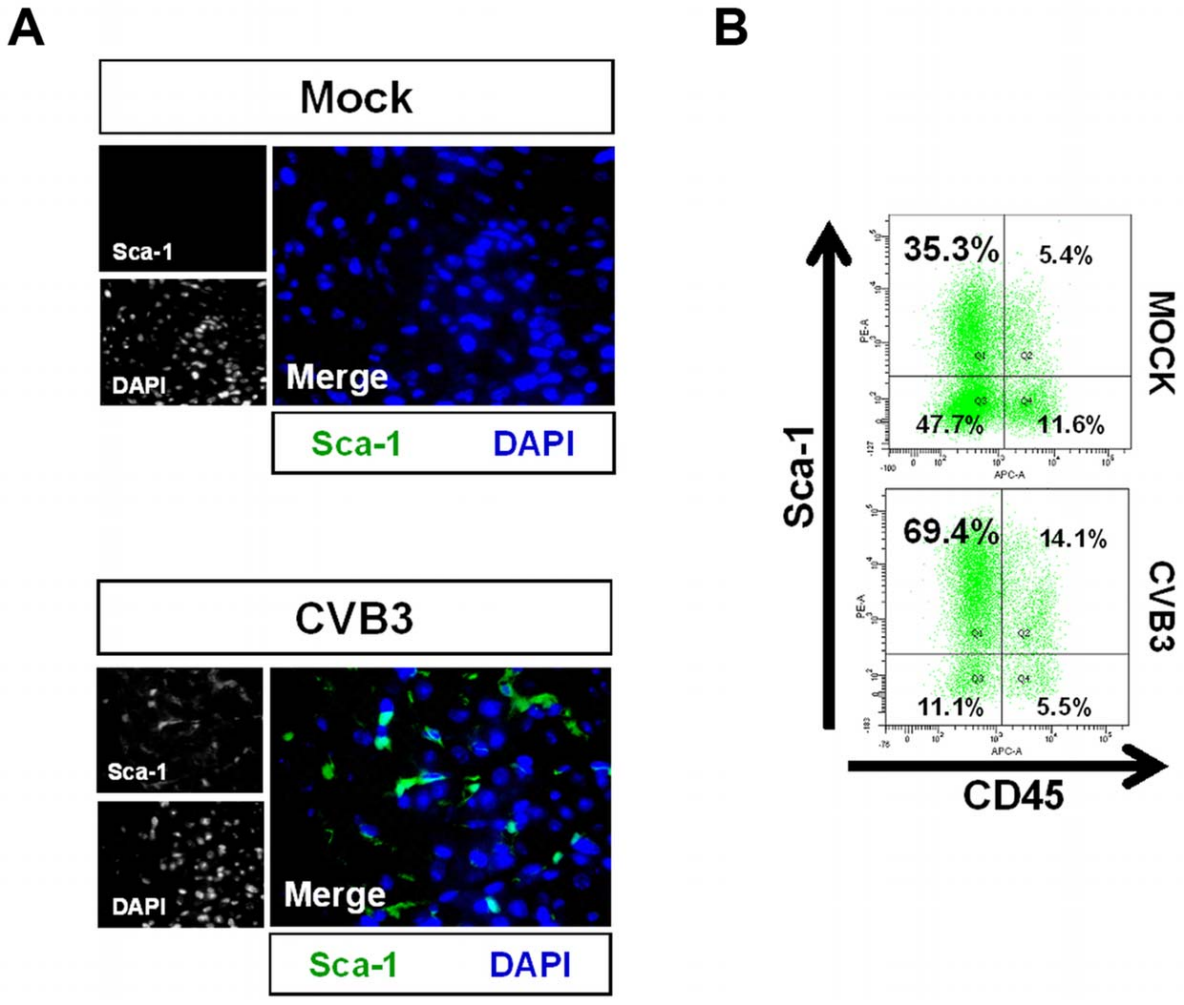
Acute CVB3 infection in juvenile mice induced an increase in myocardial Sca-1 expression. Three day-old mice were infected with eGFP-CVB3 (10^5^ pfu IP) or mock-infected, and hearts were isolated at 2 days PI. Paraffin-embedded sections of heart tissue were deparaffinized and stained using antibodies against Sca-1. Alternatively, hearts were mechanically dissociated and cells were fixed in 10% formalin in PBS for FACS analysis *(A)* An increase in Sca-1^+^ cells (green) was observed in the heart following infection. *(B)* FACS analysis confirmed an elevation of Sca-1^+^ cells in the heart following infection. Primarily resident Sca-1^+^, but also blood-derived (CD45^+^) Sca-1^+^ cells were shown to increase in number in the heart following infection. Representative flow cytometric results for three infected or mock-infected mice are shown.

To further investigate the outcome of infected CPCs, immunohistochemistry was performed on the hearts of infected mouse pups using antibodies against Sca-1 and cell lineage markers at 2 days PI (***Figure 3***). c-kit^+^ progenitor cells are thought to represent the most primitive undifferentiated population, while Sca-1^+^ progenitor cells display a transcriptional profile closer to cardiomyocytes [34], [35]. We observed a large initial increase in the number of Sca-1^+^ cells in the heart compared to mock-infected control mice (***Figure 3A***). We determined if these Sca-1^+^ cells arose from resident cardiac progenitor cells, or were instead recruited from the peripheral blood in response to viral infection. Cell suspensions derived from enzymatically dissociated infected hearts were analyzed by flow cytometry for co-expression of Sca-1 and the hematopoietic cell marker CD45. Flow cytometric results confirmed a ∼two-fold increase in the percentage of Sca-1^+^ cells isolated from the heart at an early time point following infection compared to mock-infected control mice (83.5% versus 40.7%, respectively). Expansion of Sca-1^+^ cells in the heart following infection included both CD45^+^ and CD45^−^ cell populations (***Figure 3B***). However, a greater percentage of Sca-1^+^ cells were largely negative for CD45 (69.4% versus 14.1%, respectively) or for the hematopoietic stem cell marker CD34 (31.8% versus 3.3%; data not shown), indicating the possible expansion of resident progenitor cells as opposed to the recruitment of progenitor cells having a peripheral blood origin. In combination with our histology results revealing an absence of immune infiltration, these results ruled out a significant hematopoietic cell response in the heart elicited by the virus at 2 days PI.

### CVB3-infection induced premature differentiation of cardiac progenitor cells

Though the precise role of Sca-1^+^ cell expansion in the heart following CVB3 infection remains elusive, Sca-1 protein expression has previously been shown to function in suppressing cell cycle progression [36]. Recent literature suggests that in certain precursor cell types, Sca-1 expression remains at very low levels to prevent uncontrolled cell proliferation and premature senescence. As these cells enter a differentiation program, Sca-1 upregulation leads to complete withdrawal from the cell cycle which is a necessary prequel to differentiation [36], [37]. If Sca-1 plays a similar role as a late progenitor cell marker in CPCs [34], upregulation of Sca-1 could indicate a large increase in the number of progenitor cells which quickly undergo differentiation in response to CVB3 infection.

**Figure 4:**
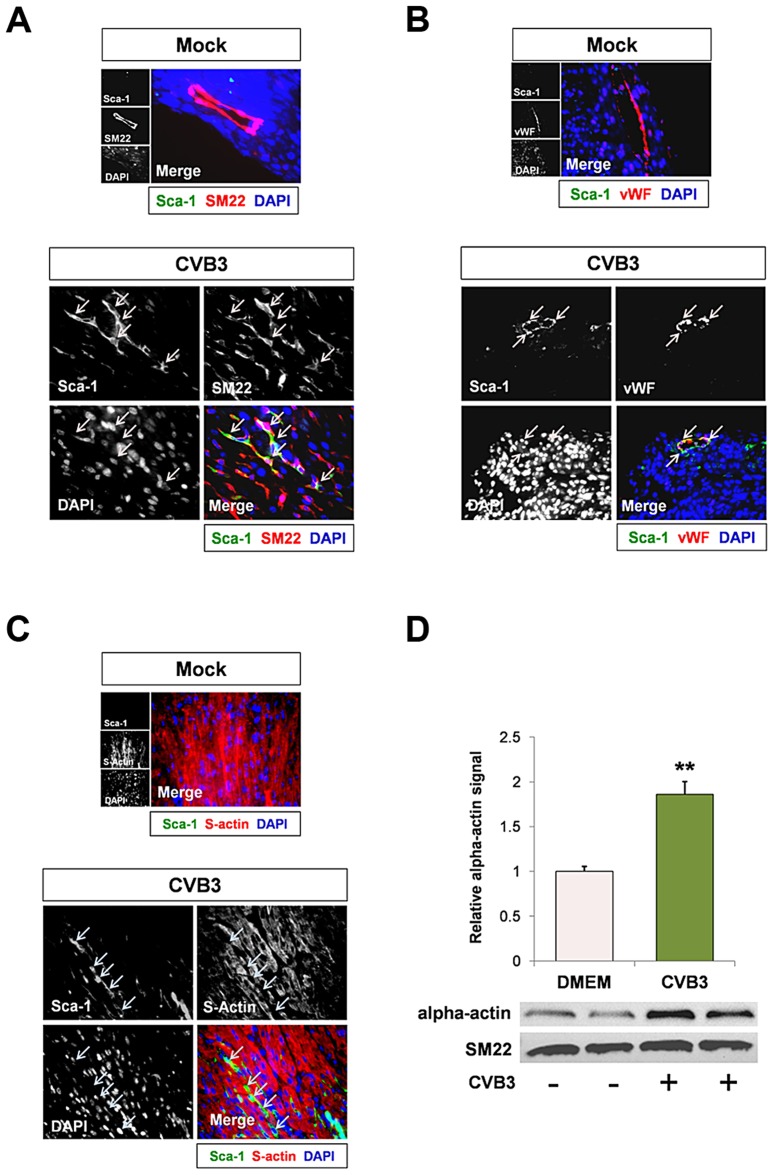
CVB3 induced premature differentiation of cardiac progenitor cells. Three day-old mice were infected with eGFP-CVB3 (10^5^ pfu IP) or mock-infected, and hearts were isolated at 2 days PI. Paraffin-embedded sections of heart tissue were deparaffinized and stained using antibodies against Sca-1, SM22, von Willebrand Factor (vWF), and sarcomeric actin (s-actin). *(A)* An increase in SM22 expression (red) was observed in infected heart sections, and Sca-1^+^ cells (green) expressed high levels of SM22 (light pink arrows). *(B)* vWF (red) was expressed near Sca-1^+^ capillaries following infection (light pink arrows). *(C)* In contrast, s-actin failed to co-localize with Sca-1 expression in the infected heart (light blue arrows). Representative images for three infected or mock-infected mice are shown. *(D)* c-kit^+^ cells were isolated from the heart of juvenile mice, expanded in culture, and infected with eGFP-CVB3 (moi = 1). Relative expression levels of alpha actin and SM22 in mock-infected or CVB3-infected juvenile c-kit^+^ cells were quantified by western analysis. A statistical significant difference was observed utilizing Student's T-test (**p<0.01).

We next inspected early expansion of Sca-1^+^ cells in the heart following CVB3 infection by identifying expression levels of cell lineage markers which colocalized with Sca-1 staining. Infected hearts were observed by immunofluorescence microscopy for Sca-1 expression and the mature cardiac lineage markers for vascular smooth muscle cells, vascular endothelial cells, and cardiomyocytes (SM22, von Willebrand factor – vWF, and sarcomeric actin, respectively). Sca-1^+^ cells showed colocalization with SM22 and vWF (***Figure 4A*** and ***Figure 4B***, respectively). However, Sca-1 did not appear to co-localize with sarcomeric actin (***Figure 4C***), suggesting possible commitment of Sca-1^+^ cells to a vascular cell lineage in the heart following infection. Intriguingly, infected c-kit^+^ cells grown in culture exhibited a sharp increase in alpha-actin expression (***Figure 4D***; **p<0.01). Also, no increase in SM22 expression was observed in isolated c-kit^+^ cells following infection, indicating that CVB3 may induce preferential cardiomyocyte differentiation in CPCs grown in culture. These results contrast with increased SM22 expression levels in infected Sca-1^+^ cells found in the mouse heart. We hypothesize that the surrounding *in vivo* microenvironment may influence cell lineage commitment patterns following CVB3 infection in the heart.

**Figure 5:**
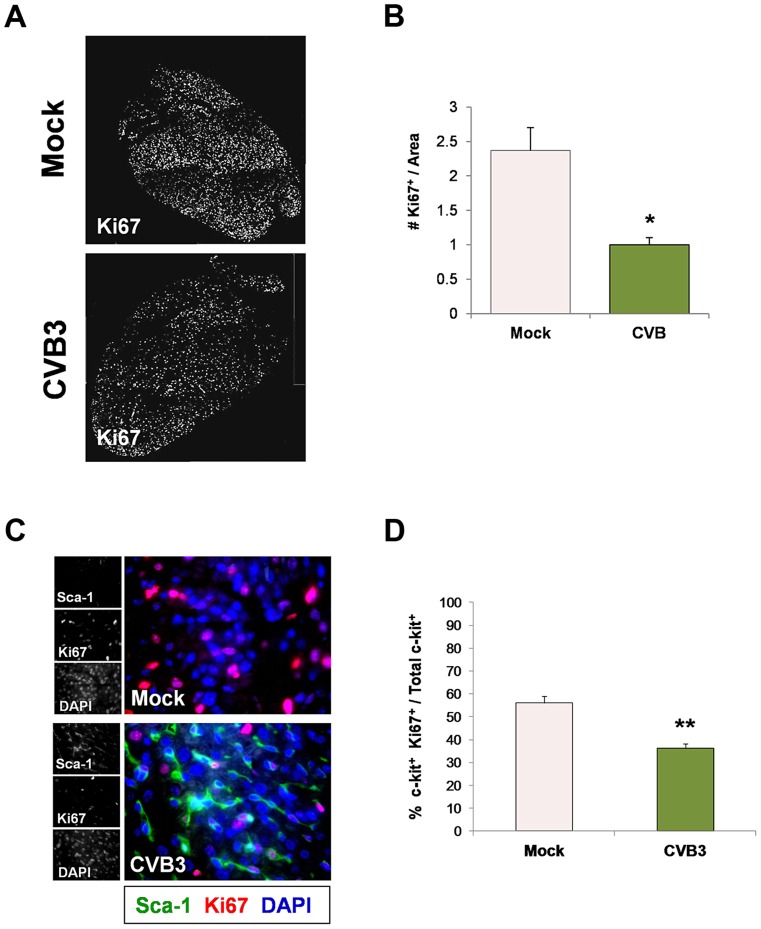
Juvenile CVB3 infection triggered growth arrest in CPCs. Three day-old mice were infected with eGFP-CVB3 (10^5^ pfu IP) or mock-infected, and hearts were isolated at 2 days PI. Paraffin-embedded sections of heart tissue were deparaffinized and stained using antibodies against Ki67, Sca-1, and c-kit. *(A)* Immunofluorescence microscopy revealed a sharp decline in Ki67^+^ cells in the neonatal myocardium at 2 days PI. *(B)* The decline of Ki67^+^ cells in the infected heart was quantified and shown to be statistically significant (*p<0.05; Student's T-test) using ImageJ software. *(C)* Immunostaining for Sca-1 and Ki67 showed a marked increase in the number of Sca-1^+^ cells in the infected heart at 2 days PI. However, these cells were predominantly negative for Ki67, indicating the lack of cellular proliferation. *(D)* Quantification of c-kit and Ki67 colocalization showed a significant decrease in the percentage of cycling c-kit^+^ cells in the heart at 2 days PI (**p<0.01; Student's T-test). Representative images for three infected or mock-infected mice are shown.

Because many viruses including CVB3 are known to alter the cell cycle, heart sections were immunostained using antibodies against c-kit, Sca-1 and the proliferation marker, Ki67. A reduction in the number of Ki67^+^ cycling cells in hearts was observed in infected animals at 2 days PI relative to mock-infected controls (***Figure 5A***), and these results were quantified by ImageJ analysis (***Figure 5B***). Although we observed an early increase in the number of Sca-1^+^ cells in the heart early after infection suggesting an initial rapid expansion of progenitor cells, Sca-1^+^ cells predominantly lacked Ki67 expression by 2 days PI consistent with its proposed role as a cell cycle suppressor (***Figure 5C***). Immunostaining for both Ki67 and c-kit revealed a significant reduction in the number of proliferating c-kit^+^ cells in the hearts of infected pups (***Figure 5D***; **p<0.01).

### Juvenile CVB3 infection depletes CPC pools in adult mice

**Figure 6:**
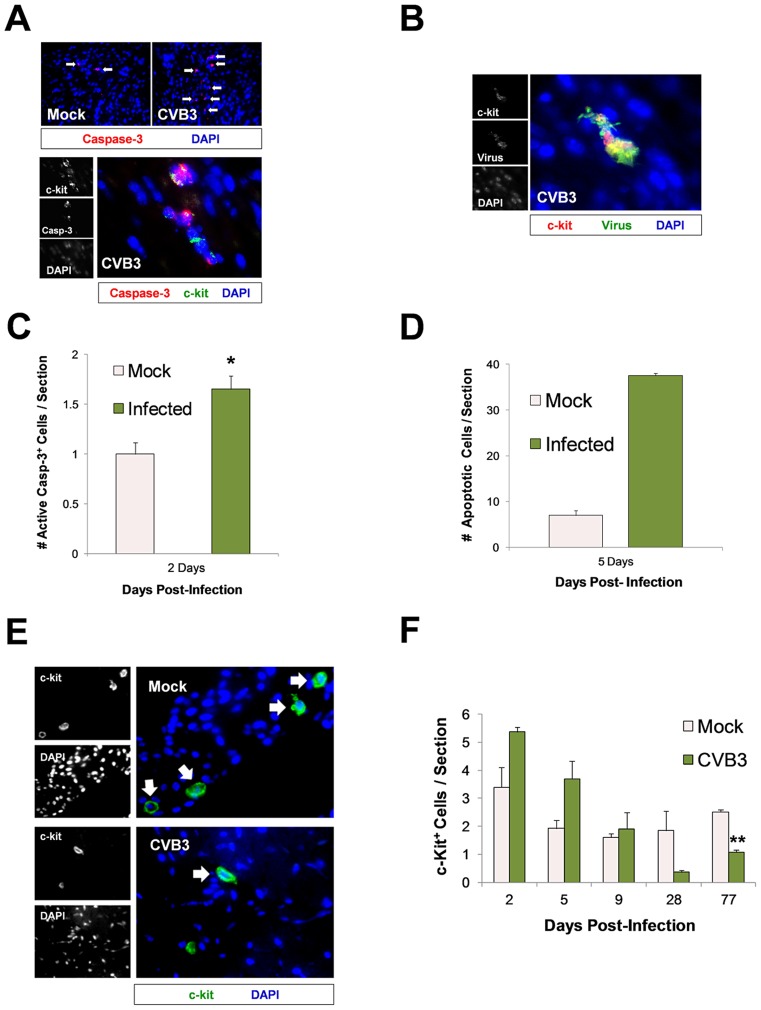
Lasting reduction of the CPC pool in the adult heart following juvenile CVB3 infection. Three day-old mice were infected with eGFP-CVB3 (10^5^ pfu IP) or mock-infected, and hearts were isolated and analyzed by immunofluorescence microscopy over time. *(A)* Deparaffinized sections were stained using an antibody against active caspase-3 (red) and c-kit (green) at 2 days PI. *(B)* Paraffin-embedded sections of heart tissue were deparaffinized and stained using an antibody against c-kit (red) and GFP (virus, green) at 2 days PI. Cellular blebbing and pyknotic nuclei were occasionally observed in infected c-kit^+^ cells. *(C)* The number of active caspase-3^+^ cells on heart sections was quantified for both infected and mock-infected mice at 2 days PI (*p<0.05; Student's T-test). *(D)* The number of TUNEL^+^ (ApopTag) cells on heart sections was quantified for infected and mock-infected mice at 5 days PI. *(E)* The number of c-kit^+^ (green) cells on heart sections was inspected at 77 days PI by fluorescence microscopy. *(F)* The number of c-kit^+^ (green) cells on heart sections was quantified over time by ImageJ analysis for both infected and mock-infected mice. A reduction in c-kit^+^ cells was observed in heart sections from infected animals at 77 days PI (**p<0.001; Student' T-test with Bonferonni correction). Pair wise comparisons were conducted using Student's T-tests to examine group differences in c-kit counts at each time point. A Bonferonni correction was applied to control for multiple comparisons, therefore the critical alpha level was set at .01. The analyses revealed that c-kit counts on Day 77 were significantly higher in the mock group compared to the CVB group, {t(4) = 11.87, p<.001}. Trend group differences also were found on Day 2 {t(4) = 2.67, p = .056}, Day 5 {t(4) = 2.49, p = .068}, and Day 28 {t(5) = 2.49, p = .055}, with c-kit counts higher in the CVB group on Days 2 and 5 but higher counts in the mock group on Day 28. The c-kit counts in each group did not differ significantly on Day 9 {t(3) = .41, p = .71}. Values were quantified from representative images taken from three infected or mock-infected mice.

CVB3 has been previously shown to induce caspase-3 dependent apoptosis in the heart and CNS [10]. To determine if CVB3 infection led to apoptosis and a permanent reduction in CPCs in the juvenile heart, sections from infected mice were immunostained using antibodies against c-kit and active caspase-3 (***Figure 6***). An increase in active caspase-3 (red) staining was observed in the infected heart, and active caspase-3 colocalized with some c-kit^+^ (green) cells (***Figure 6A***). Occasionally, infected c-kit^+^ cells in the heart showed characteristic apoptotic blebbing and pyknotic nuclei (***Figure 6B***). Quantification of active-caspase-3 staining indicated greater apoptosis in the infected heart at 2 day PI (***Figure 6C***; *p<0.05). Also, a greater number of TUNEL^+^ (ApopTag) cells per section were seen in infected hearts compared to those of mock-infected control mice at 5 days PI (***Figure 6D***). These results indicate that a significant number of cells in the infected juvenile heart which may include CPCs underwent virus-induced apoptosis.

We next determined if early CVB3 exposure resulted in a permanent decline in CPC numbers in the heart. Juvenile mice were infected with 10^7^ pfu of eGFP-CVB3, and the number of c-kit^+^ cells was evaluated in heart tissue at 2, 5, 9, 28, and 77 days PI. At 2 and 5 days PI, we observed an increased trend in the number of c-kit^+^ cells although these results did not reach statistical significance. By 77 days PI, a statistically significant decrease in c-kit^+^ cells was observed in the infected heart (***Figure 6E*** and ***Figure 6F***; **p<0.001). Sca-1, a classical cardiac progenitor cell marker which signifies progression towards differentiation, increased transiently and then remained undetectable by 5 days PI (data not shown). The observed decline in c-kit^+^ CPCs persisted despite resolution of infection by 28 days PI as shown by plaque assay for infectious virus or by qPCR for viral RNA. These results coupled with our previous findings showing that CVB3 reduced the pool of self-renewing progenitor cells in part by triggering apoptosis or premature differentiation, and progenitor cell depletion continued into adulthood despite clearance of the virus and the absence of ongoing inflammation.

### Subclinical CVB3 infection predisposed juvenile mice to beta adrenergic stress-induced heart failure later in life

Previous studies involving anthracycline-mediated cardiotoxicity suggested that juvenile exposure to the drug doxorubicin (DOX) was responsible for a profound effect on CPCs in mice, inducing their senescence and significantly reducing their number as time passed [25]. Though under basal conditions no physiological disparities or detectable cardiac deficits was observed when comparing DOX-treated mice to control animals, marked cardiomyopathy consisting of significantly increased heart mass, left ventricular dilation, and myocardial scarring was induced in DOX-treated mice following rigorous exercise via swimming. Sensitivity to stress-induced heart failure was attributed to the inability of DOX-treated animals to undergo vascular remodeling in response to the increased oxygen demand of the heart; a process which is known to be largely directed by paracrine signaling from CPCs.

**Figure 7:**
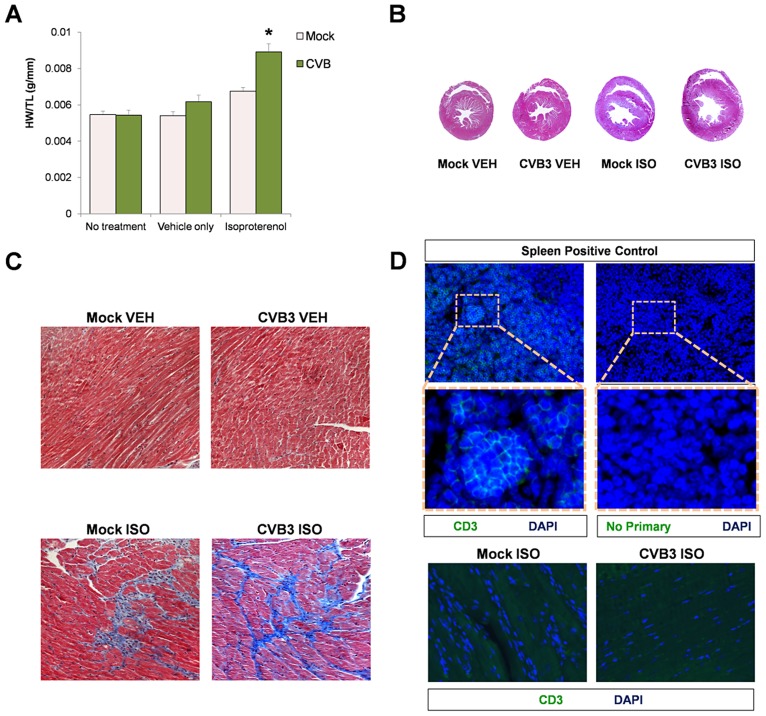
Beta-adrenergic stimulation-induced cardiac hypertrophy in adult mice infected with CVB3 at an early age leads to dilated cardiomyopathy. Three day-old mice were infected with eGFP-CVB3 (10^5^ pfu IP) or mock-infected. After 13 weeks, a 10 day treatment with isoproterenol (ISO) was used to induce physiologic hypertrophy in infected (No treatment, n = 6; Vehicle only, n = 4; ISO, n = 4) or mock-infected mice (No treatment, n = 4; Vehicle only, n = 8; ISO, n = 5). Alternatively, swimming exercise was used to induce physiologic hypertrophy in surviving mice. *(A)* Virus-infected mice showed a significant increase in heart weight to tibia length ratio following ISO treatment compared to mock-infected control mice (*p<0.001; two-way ANOVA). *(B)*, Hearts of infected mice displayed cardiac hypertrophy by H&E staining following ISO treatment. *(C)* Higher magnification of heart sections stained by Masson trichrome revealed fibrosis (blue) in both infected and mock-infected mice only after ISO-treatment. *(D)* Little to no T cell infiltration was observed in the heart following ISO treatment in both infected and mock-infected mice. A spleen positive control for CD3 staining and a No Primary antibody control are shown.

Despite a reduction in the number of CPCs following CVB3 infection, juvenile-infected mice developed normally and were phenotypically indistinguishable from mock-infected control mice at 77 days PI. We determined if stress elicited pathologic remodeling in juvenile-infected adult mice administered with the beta adrenergic agonist, isoproterenol (ISO) [38]. At 91 days PI, juvenile-infected or mock-infected mice were treated with ISO for 10 days, treated with vehicle only, or given no treatment. Although mock-infected mice showed an increase in heart weight/tibia length ratios (HW/TL) following ISO treatment, juvenile-infected animals showed a significantly greater increase in heart mass than mock-infected mice (***Figure 7A***; *p<0.001). Transverse cross sections of heart tissue from ISO-treated mice infected at an early age showed enlarged left ventricles with thinning of the muscular wall (***Figure 7B***). Also, ISO-treated mice (CVB3-infected and mock-infected) developed fibrosis, as shown by Masson's Trichrome staining (***Figure 7C***). These observations supported our hypothesis that subclinical infection with CVB3 predisposed mice to greater pathologic remodeling in the heart. Of note, no infiltrating T cells were observed in CVB3 or mock-infected heart following ISO treatment (***Figure 7D***).

### CVB3-mediated CPC loss impaired vascular remodeling during increased cardiac load

Cardiac vascular cells are highly dynamic and responsible for adaptive restructuring of the vascular network. Vascular remodeling is accomplished both through angiogenesis (branching of pre-existing blood vessels) and vasculogenesis (de novo blood vessel formation). These processes are critical for the heart to respond to various stimuli including stressors. With the induction of physiological stress, cardiac contractility increases, thereby increasing oxygen demand. Vascular remodeling is necessary to sufficiently perfuse the myocardium with oxygenated blood. Stem cells have been documented to be intimately involved in vascular remodeling through differentiation and release of paracrine factors such as vascular endothelial growth factor (VEGF) [25].

**Figure 8:**
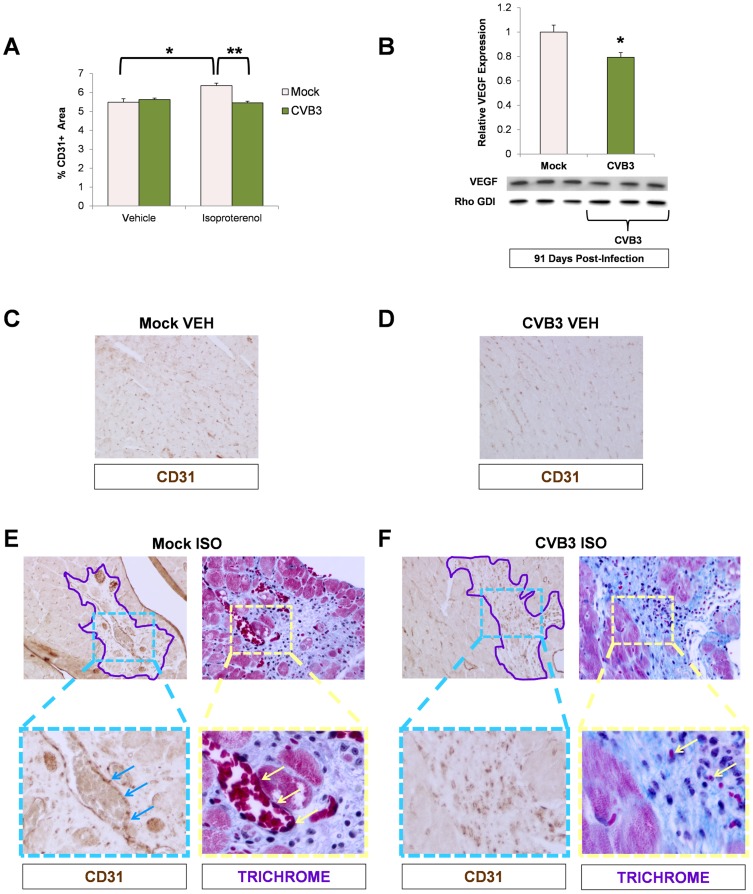
Impaired vascular remodeling following beta-adrenergic stimulation-induced cardiac hypertrophy in adult mice infected with CVB3 at an early age. Three day-old mice were infected with eGFP-CVB3 (10^5^ pfu IP) or mock-infected. After 13 weeks, a 10 day treatment with ISO was used to induce physiologic hypertrophy in infected or mock-infected mice. *(A)* Vascular changes were evaluated in the adult heart by immunostaining for CD31 (endothelial cell marker). A significant increase in CD31 staining was seen in mock-infected mice following ISO treatment (*p<0.05; two-way ANOVA). A Newman Keuls post hoc comparison test of the group×treatment interaction revealed that CD31 staining was significantly higher in the mock ISO group {6.36 (.13)} compared to the mock vehicle group {5.49 (.18)}. In contrast, a significant decrease in CD31 staining was observed in infected mice compared to mock-infected mice following ISO treatment (**p<0.01; two-way ANOVA). *(B)* A decrease in VEGF protein expression (western blot analysis) was observed in ISO-treated CVB3-infected mice (*p<0.05; Student's T-test). *(C)*, *(D)* Heart sections from vehicle only controls and immunostained for CD31 showed no differences between mock-infected and CVB3-infected mice. *(E)* CD31 staining and vascularization near sites of fibrosis (Masson trichrome staining) was observed following ISO treatment in mock-infected mice. Regions of fibrosis were outlined in light purple. CD31 staining (light blue arrows) associated with vascularization (light yellow arrows) was observed at higher magnification. *(F)* In contrast, less CD31 staining and limited vascularization was seen following ISO treatment in CVB3-infected mice.

To examine if cardiac vasculature was affected by loss of progenitor cells due to prior CVB3 infection, we stained heart sections from ISO-treated animals for the blood vessel marker, CD31. In mice treated with vehicle alone, no significant differences in CD31^+^ blood vessel density was observed between infected and mock-infected animals (***Figure 8A***). As expected, treatment with ISO in mock-infected mice resulted in increased CD31 expression consistent with increased vascular remodeling in response to the increased workload imposed by beta adrenergic stress (*p<0.05). In contrast, no corresponding increase in CD31 expression was observed in juvenile-infected mice after ISO exposure (**p<0.01). These data indicate that under increased load, the heart remodels its vasculature to meet demand, but juvenile CVB3 infection impairs this compensatory function.

Because CPCs are known to release several factors including VEGF that stimulate blood vessel formation, we next sought to determine if the production of VEGF was impaired in the heart. Western blots of heart homogenates prepared from mock and juvenile-infected mice at 91 days PI were probed for VEGF expression levels. We found that VEGF expression was significantly lower in hearts of ISO-treated CVB3-infected mice compared to mock-infected control animals (***Figure 8B***; *p<0.05). Visual inspection of heart sections in mice treated with vehicle alone showed similar levels of CD31 staining between mock-infected and CVB3-infected mice treated with vehicle alone (***Figure 8C*** and ***Figure 8D***, respectively). In contrast, high levels of CD31 staining with associated vasculature was observed in ISO-treated mock-infected mice, particularly within regions of the heart showing signs of fibrosis outlined in purple (***Figure 8E***). In CVB3-infected mice following ISO treatment, lower levels of CD31 staining and less vasculature was observed in regions of the heart showing signs of fibrosis compared to ISO-treated mock-infected mice (***Figure 8F***).

**Figure 9:**
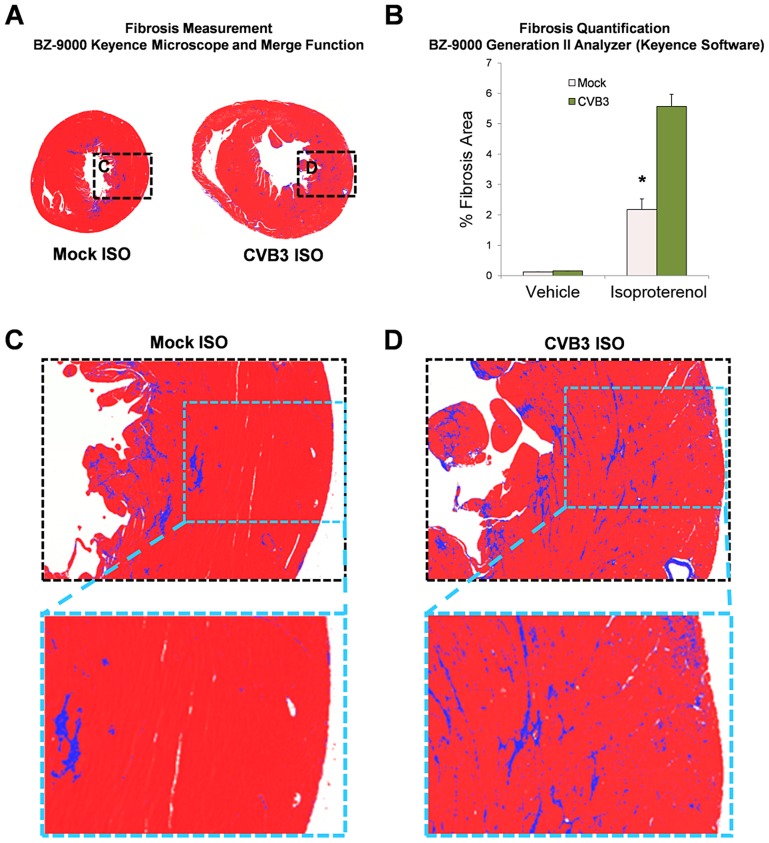
Increased fibrosis following beta-adrenergic stimulation-induced cardiac hypertrophy in adult mice infected with CVB3 at an early age. Three day-old mice were infected with eGFP-CVB3 (10^5^ pfu IP) or mock-infected. After 13 weeks, a 10 day treatment with ISO was used to induce physiologic hypertrophy in infected or mock-infected mice. *(A)* Whole heart sections were imaged with a Keyence microscope (BZ-9000) using brightfield capture to determine the degree of fibrosis (blue staining). Multiple fields were captured using the 4× objective and stitched together using the Keyence Merge function. (B) The percent fibrosis per area was quantified using the BZ-9000 Generation II Analyzer (Keyence) Single Extraction function of the Hybrid Cell Count software based on hue. A significant increase in fibrosis was observed in infected mice following ISO treatment (*p<0.001; two-way ANOVA). *(C)*, *(D)* Higher magnification of heart sections showed greater fibrosis levels in the heart of CVB3-infected mice.

The degree of fibrosis in heart sections was measured utilizing a Keyence microscope (BZ-9000) with brightfield capture in mock or CVB3-infected mice following treatment with ISO (***Figure 9***). CVB3-infected mice showed enlarged ventricles associated with greater amounts of fibrosis (blue signal) using the Keyence merge function. Fibrosis was more extensive in the hearts of CVB3-infected mice following ISO treatment compared to mock-infected mice, as quantified using the BZ-9000 Generation II Analyzer Keyence software (***Figure 9B***, ***Figure 9C*** and ***Figure 9D***; *p<0.001). These results provide further evidence that a self-limited juvenile CVB3 infection may have lasting effects on the ability of adult mice to undergo compensatory vascular remodeling under cardiac stress.

### Subclinical CVB3 infection predisposed juvenile mice to physiological stress-induced heart failure later in life

**Figure 10:**
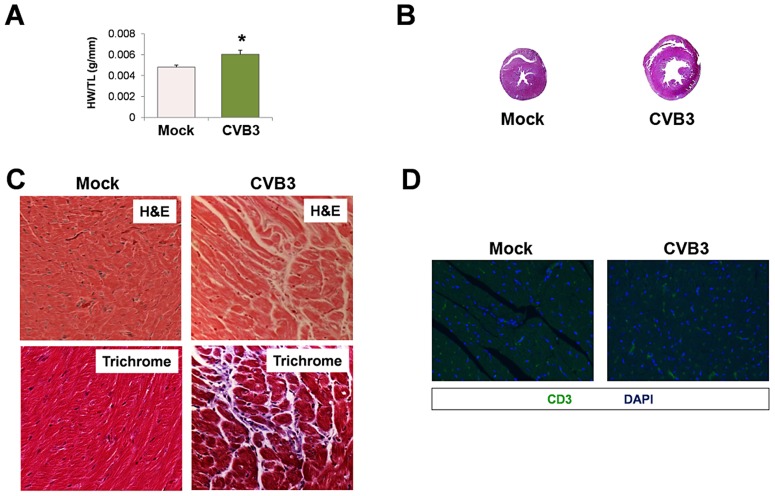
Exercise-induced cardiac hypertrophy in adult mice infected with CVB3 at an early age leads to dilated cardiomyopathy. Three day-old mice were infected with eGFP-CVB3 (10^5^ pfu IP) or mock-infected. After 13 weeks, swimming exercise was used to induce physiologic hypertrophy in surviving mice. *(A)* Virus-infected mice (n = 5) showed a significant increase (*p<0.05; Student's T-test) in heart weight to tibia length ratio following a 21 day swimming procedure compared to mock-infected control mice (n = 4). *(B)* Hearts of infected mice displayed cardiac hypertrophy by H&E staining. *(C)* Higher magnification of heart sections stained by H&E and Masson trichrome revealed signs of inflammation and fibrosis (respectively) in infected mice following the swimming procedure. *(D)* Little to no T cell infiltration was observed in the heart following the swimming procedure in both infected and mock-infected mice. Representative slide scanner images or microscopic images for three infected or mock-infected mice are shown.

Intense swimming may be considered a natural “physiologic” stress. To determine if a natural stress could induce greater heart failure following infection, adult mice infected with CVB3 at an early age were subjected to exercise challenge which consisted of 90 minutes of daily continuous swimming for 14 consecutive days. Of note, mice previously infected with CVB3 did not swim as vigorously as the mock-infected control mice (data not shown). Following the swimming protocol, we observed a significant increase in heart weight/tibia length ratios (HW/TL) in infected mice compared to mock-infected control mice (***Figure 10A***; *p<0.05). Transverse cross sections from these hearts revealed enlarged left ventricles with thinning of the muscular wall (***Figure 10B***). Also, H&E staining showed the presence of myofibrillar disarray and Masson's trichrome staining revealed fibrosis in infected mice after the exercise challenge (***Figure 10C***), although no evidence of T cell infiltration was observed (***Figure 10D***). Taken together, these data suggest that a mild juvenile CVB3 infection can result in pathologic remodeling in adult mice after exercise challenge in the absence of significant inflammation.

## Discussion

Coxsackieviruses (CV) are enteroviruses that most commonly cause a mild self-limited infection characterized by fever, upper-respiratory symptoms, and rash. In rare cases coxsackievirus B (CVB) infection can give rise to severe inflammatory diseases such as pancreatitis, meningo-encephalitis, and myocarditis. Studies of patients with end-stage idiopathic dilated cardiomyopathy have revealed detectable CVB RNA in the hearts of 70–80% of the cases, despite the absence of a history of viral myocarditis [8], [9]. This observation has raised the concern that mild CVB infection may contribute to long-term cardiac sequelae. Possible mechanisms have not been verified, although some studies have suggested autoimmune mechanisms which include superantigen expression, molecular mimicry, or bystander damage [39]. Dilated cardiomyopathy is the final common pathway of many pathologic processes, in which an enlarged ventricular cavity and thinned ventricular wall, results in poor contractility, low ejection fraction, and in many cases diastolic dysfunction due to fibrosis. These signs of disease may be preceded by many years of compensated cardiac hypertrophy characterized by thickening and enlargement of the ventricles before decompensation develops. While CVB-associated myocarditis is clearly linked to heart failure, the notion that mild CVB infection in childhood could predispose to heart failure in adults has not been previously examined. Based on our previous observation that neonatal exposure to the cardiotoxic anthracycline doxorubicin (DOX) resulted in late-onset heart failure through depletion of cardiac progenitor cells (CPCs), we hypothesized that juvenile CVB infection might reveal similar sequelae if progenitor cells in the heart were susceptible to infection similar to previous findings in the central nervous system and bone marrow [15], [17].

In this work, we established a model of mild juvenile coxsackievirus B3 (CVB3) infection which did not cause myocarditis or immune cell infiltration in the heart, but which triggered apoptosis or premature differentiation of CPCs, resulting in a significant and sustained reduction in the number of CPCs within the adult heart. Infectious virus and viral RNA were eventually cleared in the heart. We have not yet elucidated the immune mechanisms responsible for viral clearance from the heart in our juvenile mouse model. However, the limited CD3 staining in the heart observed at 2 and 77 days is consistent with previous studies demonstrating a lack of functional antiviral T cells following CVB3 infection [40], [41], and suggests that the type I interferon response may ultimately be responsible for controlling the viral infection [42]. We observed that after CVB3 exposure *in vivo*, CPCs showed a strong tendency to differentiate into vascular cells. Additionally, CVB3 has been previously shown to upregulate autophagy, a cellular process which may be essential during cellular differentiation [43]. Similar to DOX-induced senescence, CVB3-mediated premature differentiation reduced the number of CPCs available later in life. The role of CPCs in postnatal development and physiologic remodeling is poorly understood, but observations drawn from the juvenile DOX model suggest that CPCs are important for vascular development in the growing heart as well as in response to physiologic hypertrophy during exercise and in postinfarction remodeling [25]. The findings presented in this study support the hypothesis that a mild CVB3 infection early in development can impair the heart's ability to undergo physiologic remodeling, leading to dilated cardiomyopathy later in life.

Epidemiological studies have revealed an association between CVB3 infections and heart failure; however, a mechanistic link has not been found. To examine lasting consequences of CVB3 infection on the host, we infected three day-old pups with a sublethal dose of eGFP-CVB and examined hearts over time. We observed a significant decrease in the number of CPCs in the hearts of mice infected early in life. Additionally, after subjecting the mice to cardiac stress either via exercise or pharmacological induction, adult mice that had sustained juvenile CVB3 infection developed marked left ventricular hypertrophy and chamber dilation. To our knowledge, our study is the first model showing that mild neonatal CVB3 infection can predispose to pathologic remodeling in adult mice. We also observed premature differentiation of CPCs during acute infection and a sustained reduction in CPC numbers later in life despite an absence of detectable infectious virus or viral RNA in the hearts of adult animals. Also, no physiological abnormalities or cardiac damage were observed under basal conditions. Pathologic hypertrophy evidenced by left ventricular enlargement, chamber dilation, and histologic features of myofibrillar disarray and fibrosis was elicited only after adult mice were subjected to increased cardiac workload.

**Figure 11:**
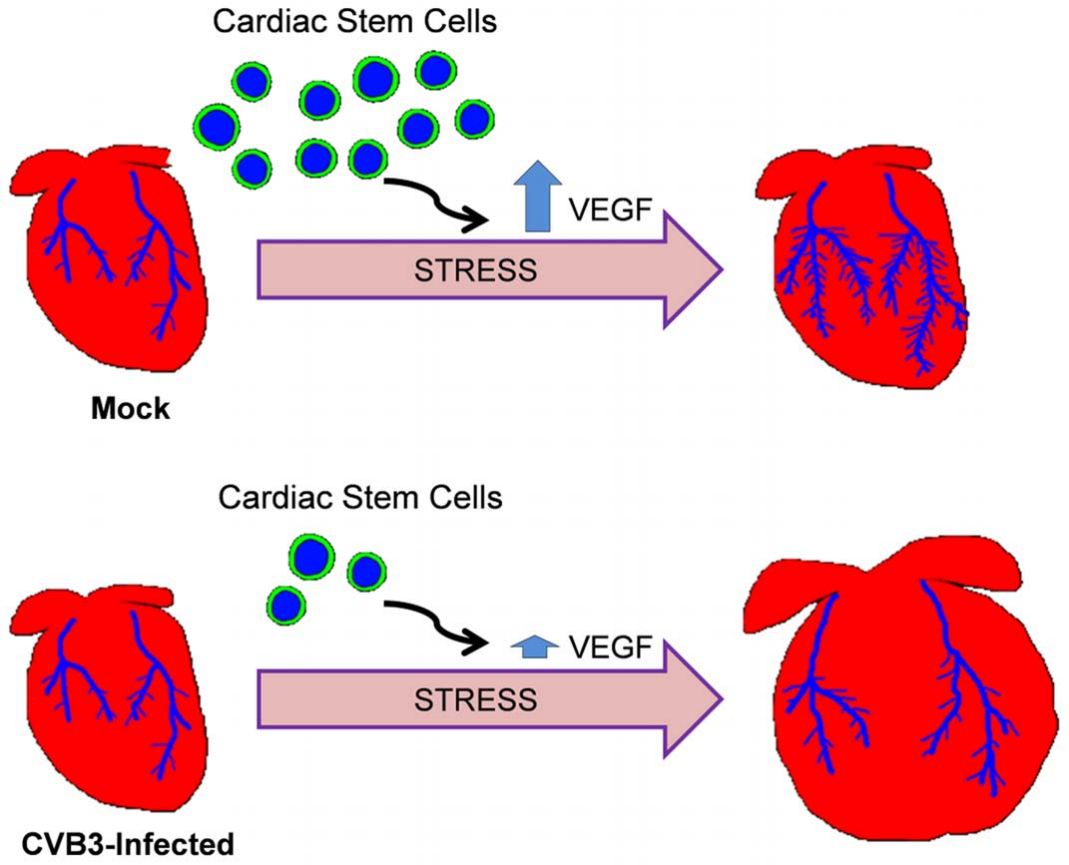
Model of adult heart failure in juvenile CVB3-infected mice. With intensified cardiac load, myocardial oxygen demand increases. CPCs are activated to stimulate angiogenesis via paracrine signaling or promote vasculogenesis via direct differentiation into vascular cells. Following juvenile CVB3 infection, CPCs are exhausted, but the heart vasculature develops normally. However, during increased cardiac load, fewer CPCs are available to participate in compensatory vascular remodeling resulting in an ischemic myocardium. This results in maladaptive ventricular remodeling.

Our model of adult heart failure in juvenile-infected mice is shown in ***Figure 11***. The propensity for pathologic alterations in the hearts of CVB3-infected mice may be due to an impairment of vascular remodeling in response to increased cardiac demand, a process which requires the participation of CPCs. Of note, endogenous c-kit^+^ cells were recently shown to substantially contribute to the generation of cardiac endothelial cells in the adult heart [44]. We did not observe differences in cardiac vascular density between mock and CVB-infected adult mice before ISO treatment. Our findings point to an impairment of compensatory remodeling rather than pre-existing deficiencies in vascular architecture. A similar phenomenon was observed following DOX-mediated CPC depletion [25]. Low dose DOX exposure in juvenile mice resulted in depletion of CPCs through early senescence. However, adult mice exhibited normal cardiac physiology and histologic findings. Stressors such as swimming exercise elicited pathologic remodeling, and experimental myocardial infarction resulted in more severe injury and poorer survival. The poor outcomes in DOX-treated mice were similarly attributed to an impaired capacity for vascular remodeling due to depleted CPCs. Stem cells have been shown to play a crucial role during vascular remodeling by stimulating angiogenesis through the release of paracrine factors, and differentiating to give rise to new blood vessels. We have previously shown that CVB3 infection of neural progenitor cells may have lasting effects on cell lineage commitment and development [45], [15]. We expect that progenitor cells in the heart surviving an early CVB3 infection may also alter CPC function and cell lineage commitment. In our studies here on the lasting consequences of juvenile CVB3 infection, a depletion or alteration of surviving CPCs which would normally participate in vasculogenesis, as well as reduced expression of the stimulatory paracrine factor VEGF in the heart likely impacted adaptive vascular rearrangement in response to cardiac stress.

These subtle cardiac alterations may go undetected under normal circumstances, but may emerge in the setting of increased cardiac demand, whether elicited by high-level exercise, chronic hypertension, or an ischemic event. Given the high prevalence of CV exposure in the population, many sedentary individuals may be asymptomatic. There are many unanswered questions that our animal model of juvenile CVB3 infection may help to address. For instance, does CVB3 infection affect stem cells at any age, or is there a high-risk window in early childhood? Anecdotally, we observed that CVB3 efficiently infected CPCs obtained from young mice (17 days post-birth), but only poorly infected CPCs obtained from adult animals. Defining the permissivity of CPCs may shed light on mechanism of pathogenesis and point to potential treatment strategies. Both permissive juvenile CPCs and non-permissive adult CPCs expressed high levels of coxsackie and adenovirus receptor (CAR). In addition, other cell types in the heart express high levels of CAR, including cardiomyocytes, without showing significant signs of infection. In contrast, some cells (B lymphocytes) lacking CAR expression are susceptible to infection [13]. The present study does not specifically define the basis for differential permissivity of cardiac progenitor cell (CPC) infection. Preferential juvenile CPC infection may be less dependent upon CAR expression and more likely determined by additional factors such as expression levels of type I interferon response genes, autophagy flux in progenitor cells, cellular differentiation, and the activation/proliferative status of these cells.

What is the fate of individual CPCs following CVB3 infection of the heart? The fate of individual CPCs infected with CVB3 would be difficult to determine without temporal cell tracking strategies. However taken as a population, our data suggest that CPCs vary in their permissivity to infection, both in terms of viral replication and cell death/survival. We have previously shown that neural progenitor cells grown in culture vary in their permissivity to infection based upon their differentiation status. CPCs grown in culture may follow a similar pattern of permissivity, although additional factors may also determine permissivity of CPCs in the heart tissue, including the host immune response.

These studies utilized a strain of CVB3 (Woodruff strain) that was obtained from a patient with myocarditis. Perhaps only some CVB3 strains pose a risk for CPC depletion and late cardiac sequelae. Due to the possible decades-long interval between childhood exposure to CV infection and development of cardiac symptomatology, challenges remain to prove causality in patients and to determine the age threshold for vulnerability. However, these studies raise concern that mild CVB3 infection in childhood may carry significant sequelae. Prevention of these late effects will depend upon introduction of a safe and effective vaccine and/or the development of rapid diagnosis and utilization of antiviral therapy in children exposed at a vulnerable age.

## Materials and Methods

### Ethics statement

This study was carried out in strict accordance with the requirements pertaining to animal subjects protections within the Public Health Service Policy and USDA Animal Welfare Regulations. All experimental procedures with mice were approved by the San Diego State University Institutional Animal Care and Use Committee (Animal Protocol Form #10-05-013F), and all efforts were made to minimize suffering.

### The generation of a recombinant CVB3 expressing the enhanced green fluorescent protein (eGFP–CVB3)

The generation of eGFP–CVB3 has been described previously [46]. Briefly, a DNA plasmid was obtained containing an infectious CVB3 clone [47] (pH 3 - kindly provided by Dr. Kirk Knowlton, University of California at San Diego). This DNA plasmid was engineered to contain an Sfi1 restriction site (pMKS1). The eGFP gene was amplified from an eGFP expression plasmid (Clontech, Palo Alto, CA) using eGFP primers with flanking Sfi1 sites. These PCR products were subsequently cloned into pMKS1. This construct was transfected into HeLa RW cells [47] (kindly provided by Dr. Rainer Wessely, University of California at San Diego) and lysates containing infectious virus were collected as viral stocks. Viral concentrations were measured via plaque assay. Viral stocks were prepared on HeLa cells and diluted in DMEM (Invitrogen, Gaithersburg, MD) before inoculation.

### Mice and viral inoculations

BALB/c mouse breeding pairs were observed daily to ensure that pups were identified within 24 hours of birth. Three day-old pups were infected intraperitoneally (IP) with 1×10^7^ pfu of eGFP–CVB3. At 2 to 10 days PI, animals were killed by hypothermia/CO_2_ asphyxiation, followed by immediate decapitation. Adult mice (4–11 weeks PI) were anesthetized with isoflurane and cervically dislocated. Hearts were removed and rinsed in cold PBS. For histology, hearts were fixed by immersion in 10% neutral-buffered formalin for 24 hours and then transferred to 70% ethanol for an additional 24 hours. The tissue was then paraffin-embedded.

### Cardiac progenitor cell (CPC) isolation and infection

CPCs were isolated using the Millipore Cardiac Stem Cell Isolation Kit (Millipore cat# SCR061) according to the manufacturer's protocol. To determine CVB3 replication in cultured CPCs, cells were seeded in 6-well plates at a concentration of 10^4^ cells/well. After 3 days, cells were infected with eGFP-CVB3 at MOI = 1.0 or 1000. Viral titers were measured in culture supernatants obtained each day following infection. For western blots, CPCs were seeded in 6-well plates at a concentration of 10^6^ cells/well. After 3 days, cells were infected with GFP-CVB3 at MOI = 100. Three days PI, cells were lysed in RIPA buffer and protein lysates were used for western blot analysis.

### Histological analysis

Paraffin-embedded hearts were cut into 4 µm thick sections. Sections were then deparaffinized with xylene and rehydrated in stepwise decreasing ethanol concentrations followed by PBS and distilled water. Antigen recovery was performed by boiling sections in sodium citrate buffer (10 mM sodium citrate+.05% Tween20, pH 6.0). For immunostaining, the following primary antibodies were used: anti-CD3 (1∶100, Biocare Med #CP215A), anti-Iba1 (1∶500, Wako #JNH4100), rabbit anti-GFP (1∶100, Invitrogen #A6455), goat anti-c-Kit (1∶40, R&D #AF1356), rat anti-Sca-1 (1∶25, Cedarlane cat #CL8934AP), mouse anti- Ki67 (1∶200, Vector #VP-K452), rabbit anti-SM22 (1∶2000, Abcam #ab14106), rabbit anti-Von Willbrand Factor (1∶100, Sigma #F3520), rabbit anti-CD31 (1∶100, Abcam #ab28364), mouse anti-sarcomeric actin (1∶100, Sigma #A2172). Perkin Elmer proprietary blocking solution (Perkin Elmer #NEL702001) was used for antibody dilution and tissue blocking. Apoptosis was detected using the ApopTag Red *In Situ* Apoptosis Detection Kit (Chemicon/Millipore, Billerica, MA) as described by the manufacturer.

### Fibrosis quantitation

Transverse sections of paraffin-embedded hearts were stained with Masson's Trichrome. Whole heart sections were imaged with a Keyence microscope (BZ-9000) using brightfield capture. Multiple fields were captured using the 4× objective and stitched together using the Keyence Merge function. Fibrosis (collagen staining) was measured using the Single Extraction function of the Hybrid Cell Count software based on hue.

### Whole heart dissociation for FACS analysis

Extracted hearts were placed in isolation buffer and excess blood was expressed by gentle compression with forceps. Hearts were then minced into ∼2 mm pieces in Millipore Isolation Buffer. Heart pieces were resuspended in Millipore Cardiac Tissue Dissociation Buffer and incubated at 37°C shaking at 200 rpm. After 45 minutes of incubation, tissue was mechanically dissociated for 5 minutes by repeated trituration with a wide-bore pipette tip. Tissues were then incubated for 15 more minutes and then mechanically dissociated for 5 minutes. Large tissue pieces were removed by filtering suspension through Millipore 100 um Steriflip unit. Cells were fixed in 10% formalin in PBS. For FACS analysis the following antibodies were used: PE-anti-Sca-1 (1∶100, BD #552108), FITC-anti-Sca-1 (1∶100, BD #557405), APC-anti-CD45 (1∶100, Caltag #MCD4505).

### Exercise challenge

Ten to eleven weeks PI, neonatally-infected mice were subjected to exercise challenge which consisted of daily swimming in heated water tanks with a gentle pump-driven current to encourage active swimming. After each swimming session, animals were allowed to recover under a heating lamp. A seven day training period consisted of 5 min swimming on day 1, then increased daily (15, 30, 45, 60, 75 and finally 90 minutes). Following this initial week of training, mice underwent 90 min daily swimming exercise for 14 days. Upon completion of 14 days of full swimming (21 day total), hearts were excised for determination of heart weight (normalized to tibia length) and histology.

### Beta-adrenergic stimulation

Isoproterenol hydrochloride was resuspended in sesame oil to prolong uptake. Thirteen weeks PI, juvenile-infected mice were injected intraperitoneally (IP) with 40 mg/kg/d isoproterenol hydrochloride daily for 10 days. Following the final day of injections, hearts were excised for determination of heart weight (normalized to tibia length) and histology.

### Statistical analyses

Statistical analyses were carried out by Student's T-test using Excel software, and by two-way ANOVA using GraphPad Prism 6 (GraphPad Software, La Jolla, CA). Significance was determined by a p-value of 0.05 or lower. Alternatively, statistical analyses were carried out by pairwise comparisons using Student's T-test with Bonferonni correction applied to control form multiple comparisons. Significance for Student's T-test with Bonferonni correction was determined by a p-value of 0.01 or lower.
