# Correction: The Impact of Juvenile Coxsackievirus Infection on Cardiac Progenitor Cells and Postnatal Heart Development

**DOI:** 10.1371/journal.ppat.1005673

**Published:** 2016-05-24

**Authors:** Jon Sin, Jenna M. Puccini, Chengqun Huang, Mathias H. Konstandin, Paul E. Gilbert, Mark A. Sussman, Roberta A. Gottlieb, Ralph Feuer

A reader has brought to our attention that there are errors in references 27, 28, and 29.

The correct reference for 27 is: Froeschle JE, Feorino PM, Gelfand HM. A continuing surveillance of enterovirus infection in healthy children in six United States cities. II. Surveillance enterovirus isolates 1960–1963 and comparison with enterovirus isolates from cases of acute central nervous system disease. American journal of epidemiology. 1966;83:455–469

The correct reference for 28 is the same as for reference 31: Kim KS, Hufnagel G, Chapman NM, Tracy S (2001) The group B coxsackieviruses and myocarditis. Rev Med Virol 11: 355–368. doi: 10.1002/rmv.326

The correct reference for 29 is: Modlin JF, Rotbart HA. Group B coxsackie disease in children. Current topics in microbiology and immunology. 1997;223:53–80

The authors confirm that all other references are listed correctly.
